# Influence of bulk nanobubble concentration on the intensity of sonoluminescence

**DOI:** 10.1016/j.ultsonch.2021.105646

**Published:** 2021-06-20

**Authors:** Toru Tuziuti, Kyuichi Yasui, Wataru Kanematsu

**Affiliations:** National Institute of Advanced Industrial Science and Technology (AIST), Moriyama, Nagoya 463-8560, Japan

**Keywords:** Amplitude, Bubble size, Laplace pressure, Blake threshold

## Abstract

The present study mainly examined the effects of the volumetric concentration of nanobubbles (ultrafine bubbles) on the intensity of sonoluminescence (SL). The addition of nanobubbles at high acoustic amplitude enhanced the SL intensity for various bubble concentrations in comparison with that in pure water. This probably means that the resulting high amplitude is over the Blake threshold, and accordingly nanobubbles expand to some extent, leading to higher SL intensity. Therefore, nanobubbles have the potential to provide nucleation sites for sonochemistry. The influence of bubble size on the intensity of SL was also evaluated.

## Highlights

• Sonoluminescence (SL) intensity was measured under different conditions of bubble concentration and acoustic amplitude.
• The addition of nanobubbles at high acoustic amplitude enhanced the SL intensity.
• Increased bubble size increased the SL intensity.

## 1 Introduction

Long-lived floating bulk nanobubbles (fine bubbles smaller than 1 μm) in liquid have recently been studied [1], [2], and the existence of gas-filled bubbles is still controversial [3], [4], [5]. However, nanobubbles have the potential to serve as cavitation nuclei in sonochemistry [6], [7], [8], [9]. To clarify the existence of gaseous nanobubbles, it is necessary to distinguish them from solid or liquid particles [10]. Recently, Hata et al. observed sonoluminescence (SL) [9], [11], [12], [13], [14] in water containing argon nanobubbles to find that SL evidently occurred in the presence of nanobubbles compared with that in their absence [15]. SL is defined as light emission from a cavitating liquid exposed to intense ultrasound [9]. Tuziuti et al. measured the intensity of SL for various acoustic amplitudes in both the presence and absence of air nanobubbles to show that at an high amplitude, where a decrease in SL intensity is often observed, the SL intensity in the presence of nanobubbles could be higher than that when they are absent [16], [17]. These results imply that SL is an indicator to evaluate the existence of nanobubbles in a liquid.

However, to the best of our knowledge, to date only minimal research has focused on the intensity of SL when the concentration of nanobubbles is varied. The present study mainly deals with measurement of the SL intensity at different concentrations of nanobubbles and evaluates incremental increases in the intensity by nanobubbles compared with that in their absence. Recently, the authors reported preliminary assessments of the SL intensity at different concentrations of nanobubbles [18]. In this prior work, the SL intensity at different volumetric concentrations of nanobubbles was monitored to obtain the ratio of SL intensity in the presence of nanobubbles against that in the absence of nanobubbles at high acoustic amplitude. It was found that the ratio was higher than 1 for different volumetric concentrations and the possible mechanism was discussed.

The present study examined the intensity of SL for various concentrations of nanobubbles as a function of acoustic amplitude, as well as the intensity in the absence of nanobubbles, while varying a greater number of parameters than in our previous work [18]. Primarily, a normalized SL intensity, the ratio of intensity in the presence of nanobubbles against that in their absence at high acoustic amplitude, as a function of bubble volumetric concentration was evaluated to clarify the influence of nanobubbles on the SL intensity. The ratio per nanobubble as a function of bubble size was also discussed.

## 2 Materials and methods

Nanobubbles were prepared in pure water (Millipore Essential Elix 5) using a bubble generator apparatus (YBM Faby-10) that uses the impact of bubble cavitation and the shear force imparted by vortex flow, as described in a previous publication [19]. The concentration of nanobubbles was adjusted by mixing nanobubble water with air-saturated pure water at different ratios for dilution. The air saturation was established by bubbling air at 23 °C in a thermostatic chamber (TAITEC BR-40LF).

The size of nanobubbles was measured with a size distribution measurement apparatus (Shimadzu SALD-7500X10). The principle of size measurement using this apparatus has been described elsewhere [20], [21]. The size of particles (bubbles) was obtained by software (Shimadzu WingSALD II). The term “concentration” used in the present paper means the total concentration value that was obtained by integrating the bubble concentrations over the entire distribution.

The SL intensity-measurement procedure is described in [17]. The SL intensity in the water in the presence and absence of nanobubbles during exposure to 54-kHz continuous-wave sinusoidal ultrasound was measured for various acoustic amplitudes. The driving frequency (54 kHz) is low for sonochemical reaction [22], however, relatively suitable for obtaining large expansion of a bubble leading to high intensity of SL [23] at compression phase of an ultrasound. A rectangular glass cell (inner dimensions: 50 × 50 × 140 mm) was filled with 100 mL of nanobubble water, and a resonant standing wave field was created in the longitudinal direction between the liquid surface and the cell bottom, which was open and faced the surface of the vibration plate on the driving transducer. The transducer diameter was 45 mm. The SL emitted from the entirety of the liquid volume was detected using a photomultiplier tube (PMT) and adjustable lens system. The signal amplitude given to the power amplifier driving the ultrasonic transducer was varied from 0 to 500 mV (peak-to-trough) in increments of 5 mV, with SL intensity data collection over a span of 38 s. Intermediate concentrations of nanobubbles were adjusted by mixing water containing nanobubbles as prepared by the bubble generator with water without nanobubbles.

## 3 Results and discussion

**Fig. 1:**
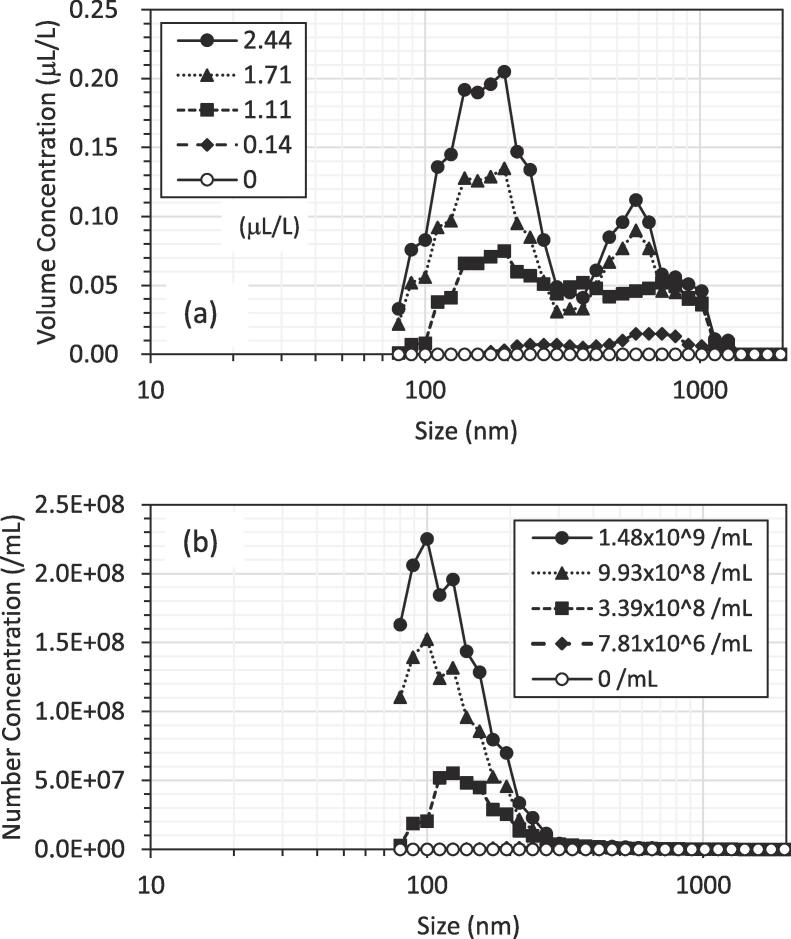
Size distribution of nanobubbles at different concentrations plotted as (a) volume and (b) number representation.

Fig. 1 shows the size distribution of nanobubbles at different concentrations plotted as the volume and number representation, respectively. Each plot is the average of three data points. In both representations, each of the distributions, including the distributions at the intermediate diluted concentrations, has a value mainly in the range of 100–200 nm, which agrees with that in the literature [24]. Note that the distribution for the number concentration at 7.81 × 10^6^ /mL is close to zero in Fig. 1b. In both representations, the size distribution decreases with the extent of dilution. This is because the concentration of nanobubbles for the measured liquid decreases with the extent of dilution. Moreover, as the dilution increases, the component of the smaller size decreases to more than that of the larger size. This probably is because the smaller bubbles dissolve more easily due to the Laplace pressure and the increased dilution makes the bubbles become less stable. Note that since the component of larger size did not necessarily increase with dilution, Ostwald ripening for growth mechanism of nanoparticles [25] meaning that smaller particles dissolve into surrounding liquid leading to the disappearance to cause larger ones to grow would not work. According to the literatures [5], [26], [27], bulk nanobubbles probably disappeared by interaction with the free surface of the liquid or the wall of the container. This has been corroborated recently by Kanematsu et al. [10]. The operation of dilution causes mixing liquid leading to the increase in the chance of contact of bubbles to both the liquid surface and the liquid container compared with that without dilution.

As for the temporal stability of the bubble size, the authors confirmed at least that the deviation of the average bubble size of nanobubble water without dilution but prepared in the same way as the present method stayed within 4% after 17 min passed, which was longer than the time required for each of the present measurements.

**Fig. 2:**
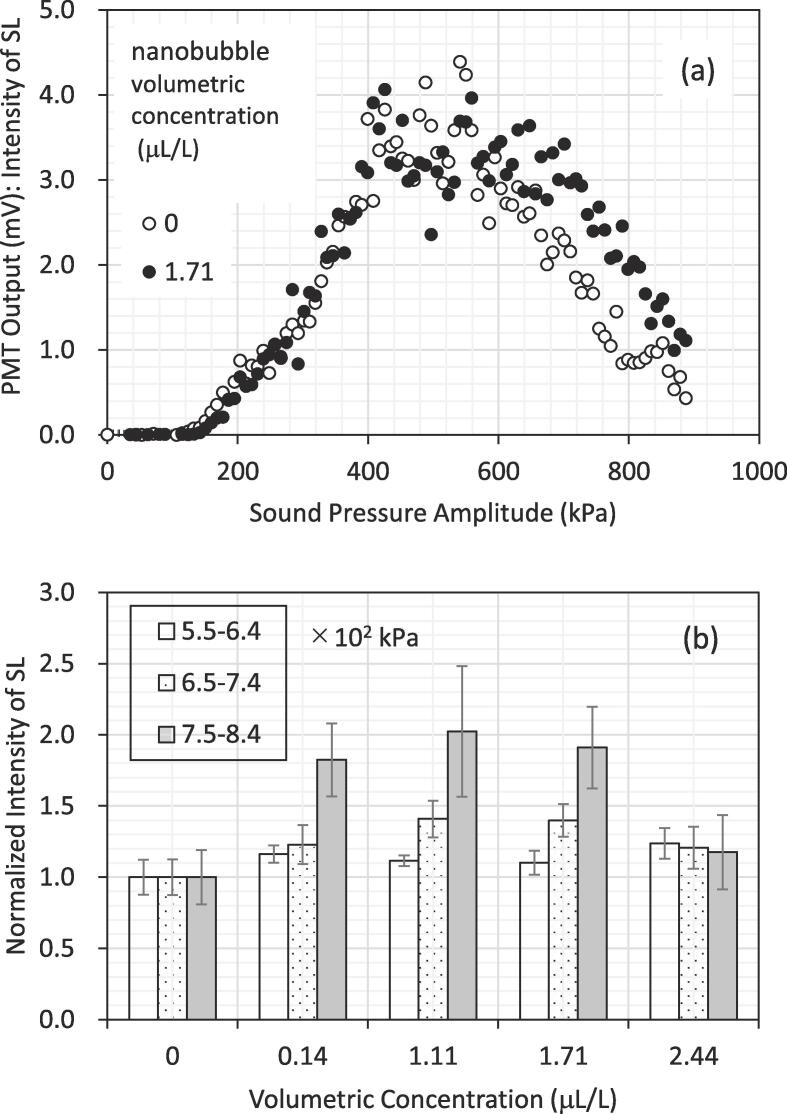
Sound-pressure amplitude dependence of the average value of SL intensities: (a) Example of intensity of sonoluminescence (SL) in the presence and absence of nanobubbles as a function of sound pressure amplitude, (b) the SL intensities of four nanobubble concentrations normalized by the SL in the absence.

Fig. 2 shows sound-pressure amplitude dependence of the average value of SL intensities. Fig. 2a shows an example of SL intensity in the presence and absence of nanobubbles as a function of sound pressure amplitude. Each plot is the average of four data points. Note that, the sound pressure amplitude at the FG output voltage up to 500 mV peak-to-trough was extrapolated on the basis of the linearity obtained between the FG output voltage and the sound pressure amplitude measured at low FG output voltage ranged from 0 to 25 mV peak-to-trough for both pure water and water containing nanobubbles using a calibrated hydrophone (Brüel & Kjær 8103) set at the antinode nearest the liquid surface on the sound beam axis [17]. As the pressure amplitude increases, the SL intensity of both cases increases to the maximum. After that, the SL intensity decreases at increased pressure amplitude. This decrease in SL intensity at excess pressure (quenching) is often observed [28] and comes from both the expulsion of bubbles from the pressure antinode by action of Bjerknes force at excess amplitude [28] and the excitation of liquid surface vibration to break the resonance of the standing wave [29]. In Fig. 2a, when the sound pressure is less than 5 × 10^2^ kPa, there is little difference in SL intensity with and without nanobubbles. This is probably because at relatively low amplitude it is hard for nanobubbles to pulsate enough to emit SL by overcoming the restriction due to surface tension of the surrounding liquid. Therefore, an effect of adding nanobubbles on SL intensity did not occur at relatively low amplitude. However, when the sound pressure becomes 5 × 10^2^ kPa or more and the quenching phenomenon appears, the SL intensity with nanobubbles is higher than that without nanobubbles. The existence of nanobubbles has an effect of inhibiting the quenching since large-sized bubbles are expelled from the region of high-pressure amplitude while submicron-sized bubbles could stay there to cause pulsation leading to the emission of SL. In order to inhibit the quenching of SL, Hatanaka et al. [30] made relatively small bubbles to stay at the region of high-pressure amplitude by maintaining the distance between bubbles leading to suppression of bubble–bubble coalescence by external forced fluid flow.

The difference in the intensity between the absence and presence of nanobubbles was observed at the amplitude of more than 6 × 10^2^ kPa. To find the difference clearly, the ratio of the SL intensity in the presence of nanobubbles against that in their absence at each pressure amplitude was calculated and the average over the range of relatively high amplitude of more than 6 × 10^2^ kPa was obtained for different concentrations of nanobubbles. According to the results of numerical calculations reported in the literature [23], a greater SL energy value is associated with a high amplitude of 6 × 10^2^ kPa compared to the value obtained with a lower amplitude of 1.75 × 10^2^ kPa when producing nanobubbles at a relatively low ultrasonic frequency of 20 kHz. This is because the higher amplitude could be over the Blake threshold [9] and bubbles were pulsing to emit SL effectively. Thus, the present range of sound pressure amplitude to evaluate the ratio was limited to the range of around 6 × 10^2^ kPa or more.

An influence of the concentration of nanobubbles on the relationship between sound pressure amplitude and SL intensity was investigated. Fig. 2b shows the normalized SL intensity, defined as the average ratio of SL intensity in the presence of nanobubbles for the range of around 6 × 10^2^ kPa or more in sound pressure amplitude compared with the intensity in the absence of nanobubbles. Fig. 2b is for different volumetric concentrations of nanobubbles. For each pressure amplitude range, the obtained SL-intensity data of about 40 points were averaged. The normalization of SL intensity in the presence of nanobubbles for different concentrations was made against that in their absence. As shown in the figure, the ratio is higher than 1 at each of the concentrations in the presence of nanobubbles. Commercial software (Excel Microsoft 365) was used to statistically analyze the SL intensity difference between nanobubble presence and absence; the p-value was 2.6% at most. If a statistical significance level of 5% is chosen, the p-value of 2.6% is less than 5% and should be sufficient. This probably means that the given high amplitude is over the Blake threshold, and accordingly nanobubbles could expand to some extent, leading to SL, where the Blake threshold is the condition for large expansion of a bubble [9]. It was found that the ratio becomes low at a high concentration (2.44 μL/L). This may be due to shielding of bubbles, which makes for ineffective propagation of sound to the inner bubbles in bubble clusters less than the resonant size caused by primary and secondary Bjerknes forces, leading to SL suppression [11], [31].

**Fig. 3:**
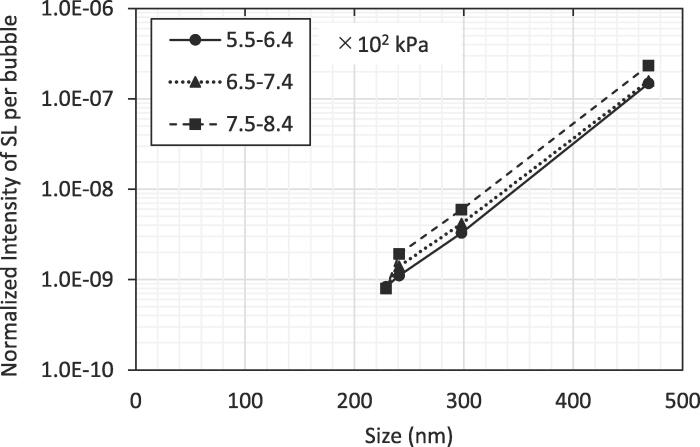
Normalized SL intensity per bubble as a function of nanobubble volumetric size for 5.5–6.4 × 10^2^, 6.5–7.4 × 10^2^, and 7.5–8.4 × 10^2^ kPa in sound pressure amplitude.

Fig. 3 shows the per-bubble normalized intensity as a function of nanobubble volumetric size, where each volumetric size in the horizontal axis is the average over the whole corresponding distribution in the presence of nanobubbles in Fig. 1a. That is, the volumetric sizes of 229, 241, 298, and 469 nm in Fig. 3 are for 2.44, 1.71, 1.11, and 0.14 μL/L in Fig. 1a, respectively. The volumetric size represents the weighed averaged diameter with volumetric concentration. The normalized intensity per bubble, the ratio (SL intensity compared with that in the absence of nanobubbles appeared in the longitudinal axis in Fig. 2b) divided by the total number concentration of bubbles, is taken for the longitudinal axis for Fig. 3, where the total number concentration value that was obtained by integrating the bubble number concentrations over the entire distribution in Fig. 1b and means the number of bubbles in 1 mL. The ratio per bubble typically increased with bubble size for different amplitudes. This probably means that the larger bubble size enabled bubbles to expand more by overcoming the limitation of bubble expansion due to the surface tension of the surrounding liquid. Therefore, violent collapse at compression was attained, leading to high SL intensity.

## 4 Conclusions

Through the measurement of bubble size distribution and SL intensity under different conditions of bubble concentration and acoustic amplitude, it was clarified that the addition of nanobubbles at high acoustic amplitude enhanced the SL intensity for various bubble concentrations in comparison with that in pure water, and the increased bubble size provided higher SL intensity. These results might come from the gaseous nature of nanobubbles. The significance of the results was that the existence of nanobubbles under sonication could inhibit sonoluminescence quenching at high amplitude and provide cavitation nucleation sites useful for yield enhancement of sonochemical reaction. Such effects by nanobubbles could not be obtained at low amplitude because of the restriction due to surface tension by the surrounding liquid.

## Declaration of Competing Interest

The authors declare that they have no known competing financial interests or personal relationships that could have appeared to influence the work reported in this paper.
